# Chemotherapy and radiation therapy elicits tumor specific T cell responses in a breast cancer patient

**DOI:** 10.1186/s12885-016-2625-2

**Published:** 2016-08-03

**Authors:** David Bernal-Estévez, Ramiro Sánchez, Rafael E. Tejada, Carlos Parra-López

**Affiliations:** 1 Immunology and Traslational Medicine Research Group, Graduated School in Biomedical Sciences, Department of Microbiology, Medical School, Universidad Nacional de Colombia, Carrera 30 #45-03 Building 471, office 304, Bogotá, Colombia South-America; 2 Immunology and Clinical Oncology Research Group (GIIOC), Fundación Salud de los Andes, Calle 44 No. 58-05, Bogotá, Colombia South-America; 3 Clínica del Seno, Carrera 11 # 68-36, Bogotá, Colombia South-America; 4 Hospital Occidente de Kennedy E.S.E., Servicio de Oncología, Bogotá, Colombia South-America; 5 Facultad de Medicina, Departamento de Microbiología, Universidad Nacional de Colombia, Carrera 30 Calle 45, Bogotá, Colombia

**Keywords:** Breast cancer, Type I alpha dendritic cells, T cells, Chemotherapy, HER2/neu, CTLA-4, TCR repertoire

## Abstract

**Background:**

Experimental evidence and clinical studies in breast cancer suggest that some anti-tumor therapy regimens generate stimulation of the immune system that accounts for tumor clinical responses, however, demonstration of the immunostimulatory power of these therapies on cancer patients continues to be a formidable challenge. Here we present experimental evidence from a breast cancer patient with complete clinical response after 7 years, associated with responsiveness of tumor specific T cells.

**Methods:**

T cells were obtained before and after anti-tumor therapy from peripheral blood of a 63-years old woman diagnosed with ductal breast cancer (HER2/neu+++, ER-, PR-, HLA-A*02:01) treated with surgery, followed by paclitaxel, trastuzumab (suspended due to cardiac toxicity), and radiotherapy. We obtained a leukapheresis before surgery and after 8 months of treatment. Using in vitro cell cultures stimulated with autologous monocyte-derived dendritic cells (DCs) that produce high levels of IL-12, we characterize by flow cytometry the phenotype of tumor associated antigens (TAAs) HER2/neu and NY-ESO 1 specific T cells. The ex vivo analysis of the TCR-Vβ repertoire of TAA specific T cells in blood and Tumor Infiltrating Lymphocytes (TILs) were performed in order to correlate both repertoires prior and after therapy.

**Results:**

We evidence a functional recovery of T cell responsiveness to polyclonal stimuli and expansion of TAAs specific CD8+ T cells using peptide pulsed DCs, with an increase of CTLA-4 and memory effector phenotype after anti-tumor therapy. The ex vivo analysis of the TCR-Vβ repertoire of TAA specific T cells in blood and TILs showed that whereas the TCR-Vβ04-02 clonotype is highly expressed in TILs the HER2/neu specific T cells are expressed mainly in blood after therapy, suggesting that this particular TCR was selectively enriched in blood after anti-tumor therapy.

**Conclusions:**

Our results show the benefits of anti-tumor therapy in a breast cancer patient with clinical complete response in two ways, by restoring the responsiveness of T cells by increasing the frequency and activation in peripheral blood of tumor specific T cells present in the tumor before therapy.

**Electronic supplementary material:**

The online version of this article (doi:10.1186/s12885-016-2625-2) contains supplementary material, which is available to authorized users.

## Background

Nowadays exists clinical evidence that the quality and number of tumor infiltrating lymphocytes (TILs) is crucial for clinical outcome of cancer patients [1]. On the other hand, Kroemer and colleagues have published abundant experimental evidence that suggests that some chemo-radiotherapy regimens in cancer generate T cell stimulation that accounts for the clinical response induced by these therapies [2]. Despite all this experimental evidence, the demonstration of the stimulatory power of the anti-tumor therapy (anti-TTx) on tumor-specific T cells in cancer patients with complete response after cancer treatment continues to be a formidable challenge. The anergy induced early in the course of the tumors [3], followed by tolerance and exhaustion of T cells occurring late in a wide variety of solid tumors [4], together with the low frequency of T cells specific for tumor associated antigens (TAAs) [5, 6] fosters the unresponsiveness of T cells to tumor antigens that is one important hurdles to monitor the response of T cells specific for TAA in cancer patients during anti-TTx. The design of in vitro systems that circumvent these obstacles and that leads us into immunological readouts useful to monitor the response of anti-tumoral T cells during cancer treatment, may become instrumental in establishing correlates between tumor outcome achieved with these therapies and the response of T cells to tumors in vivo. CD8+ T cells in peripheral blood are themselves a complex mixture comprised of at least four major subsets – naïve (TN), central memory (TCM), effector memory (TEM) and effector memory expressing CD45RA (TEMRA) subsets – each having different functional qualities [7]. Efforts have focused on identifying traits of T cells in vitro that correlate with anti-tumor responsiveness in vivo. TEMRA cells are developed from long-lasting memory cells and because their potent effector cytolytic capacity they are the responsible for tumor control. It is expected that evidencing the development of TEMRA cells in vitro may be a reflection of the generation of proficient anti-tumor immunity in vivo [8, 9].

Geiger and colleagues attempted to overcome the limited detection capacity of functional T cell assays for the detection of naïve and memory antigen (Ag)-specific T cells in blood through the augmentation of the number of these cells using in vitro expansion, however, this approach is time consuming and requires considerable manipulation [10]. To overcome these limitations, we consider that the use of dendritic cells (DCs) induced and matured *in situ* as antigen presenting cells (APCs), using the standard maturation cocktail (stDCs) [11] or the cytokine mix recently described for the induction of Type I alpha DCs (aDCs) characterized by the production of high levels of IL-12 [12] to activate memory T cells [13] or to prime in vitro naïve T cells present in peripheral blood mononuclear cells (PBMCs) [14], might be a powerful approach for measuring the response of tumor-specific T cells that expand in cancer patients in response to anti-TTx. In search for in vitro assays that helps to establish a correlation between clinical tumor outcome and T cell responses elicited by anti-TTx in cancer patients, we performed a series of functional assays with T cells obtained from a breast cancer patient before and after anti-TTx that were stimulated in vitro with two types of DCs pulsed with TAAs.

Our results suggest that the stimulation of T cells with Type I alpha DCs derived in two days (2d-aDCs) pulsed with TAAs allowed us to demonstrate that anti-TTx rescues T cells from the profound unresponsiveness status typically observed in patient T cells before treatment, this recovery of T cell function could be explained in part by the production of IL-12 by 2d-aDCs (data not show). The T cell responsiveness after anti-TTx was reflected in the recovery of TCR internalization; expression at the cell surface of T cell activation markers; activation of effector T cells specific for several TAAs and in the expansion in peripheral blood of T cells specific for TAAs that were present in the tumor infiltrate prior anti-TTx.

## Methods

### Patient and volunteers PBMCs isolation

This study was approved by the ethics committee of the Medical School – Universidad Nacional de Colombia (CE-14, 9 August 2008, Act. 107). The patient MCC-002 and all healthy donors signed an informed consent form before blood samples were taken. Heparinized blood samples were obtained from healthy volunteers (60 mL). From patient MCC-002 a leukapheresis was obtained before and after eight months of having finished the treatment (Additional file 1: Figure S1). PBMCs were purified using density gradient centrifugation with Ficoll-Paque PLUS (GE Healthcare Life Sciences) and cryopreserved in freezing medium containing 50 % RPMI-1640 + 40 % fetal bovine serum (FBS) (Gibco - Life Technologies) + 10 % Dimethyl sulfoxide (DMSO) (Sigma-Aldrich, St. Louis, United States) at a maximum concentration of 10^7^ cells/mL using controlled freezing temperature with an isopropanol filled container and afterwards stored in liquid nitrogen until use. The viability of cells was evaluated directly with 0.4 % Trypan Blue (Life Technologies) and/or with flow cytometry (FC) using LIVE/DEAD Fixable Aqua Dead Cell Stain Kit (Life Technologies).

### T cell purification

CD4+ and CD8+ naïve T cells were purified using MACS cell separation with Naïve T CD4+ Cell Isolation Kit II and Naïve CD8+ Cell Isolation Kit (Miltenyi Biotec, Germany) system with magnetic labeled antibodies following manufacturer’s protocol, briefly PBMCs were resuspended in MACS buffer (RPMI-1640 + 0.5 mM EDTA + 1 % FBS) and labeled with corresponding antibody cocktail, after cells were washed in MACS buffer, they were pass-through in a humidified (MS) column. Positive cells were washed twice and cell purity was verified by flow cytometry with a purity > 95 %.

### T cells stimulation

Two different T cell stimulation methods were used (Additional file 2: Figure S2). Total PBMCs were enriched with 2d-stDCs or 2d-aDCs based on the methodology of Martinuzzi et al., [13]. Briefly, 10^6^ PBMCs were cultured with IL-4 and GM-CSF as described by Scandella [15] in the presence of 5 μM of tumor-associated antigens (TAAs), HLA-A*02:01 restricted viral peptides (CMV, FLU or EBV) for 24 hours and subsequently maturated using either 2d-aDCs or 2d-stDCs maturation cocktails [12, 16], with the addition of 5 μM of the corresponding peptide(s) for 6 days (Additional file 2: Figure S2A); in some experiments unpulsed DCs were used as control in order to determine the basal level of T cell stimulation. The second method using purified CD4+ or CD8+ naïve T cells is based on the methodology published by Moser et al., [14]. Briefly, purified monocytes were differentiated into 2d-stDCs or 2d-aDCs, pulsed or unpulsed with TAA peptide(s) (5 μM each) and subsequently cultured with purified CD4+ or CD8+ naïve T cells for 14 days at a ratio of 50:1 (T cell: DCs) in serum free AIM-V culture media (Life Technologies, Carlsbad, CA, United States). After 14 days of priming, CD4+ or CD8+ T cell cultures were boosted with corresponding peptide-pulsed 2d-stDCs or 2d-aDCs and cultured for 6 additional days (Additional file 2: Figure S2B). For polyclonal stimulation, 5×10^6^ PBMCs/mL were stimulated in different conditions; (i) 2d-aDCs induced in PBMCs *in situ*; (ii) combining 2d-aDCs with T cell activation/Expansion kit (antiCD3/CD28/CD2 micro beads - Miltenyi Biotec) for 24 h after differentiation of 2d-aDCs; (iii) only with T cell activation/Expansion kit; and (iv) PBMCs cultured without stimulation for 3 days as control.

### Flow cytometry and cytokine quantification

For the staining procedure, cells were collected and stained in a final volume of 50 μL of staining buffer (PBS + 1 % FBS) for 30 min at 4 °C and washed with staining buffer before flow cytometry acquisition. Purified T cells or total PBMCs were stained with the following fluorescent antibodies: CD3, CD4, CD152 (CTLA-4), BTLA, PD1 or CD69 (Biolegend, San Diego, United States) or CD8 (eBiosciences, San Diego, United States), CD45RO, CD45RA, CD62-L (BD), CD154 (CD40L) (eBiosciences, San Diego, United States), CD95 (Fas), or CCR7 (R&D Systems). Cytokine secretion (TNF-α, IFN-γ, IL-6 or IL-12p70) was measured in the culture supernatants using human Th1/Th2 and Inflammatory CBA kits (BD Biosciences) following manufacturer’s protocol. All samples were acquired using the FACSAria II (BD Biosciences) at the Universidad Nacional de Colombia; cytometric bead array (CBA) data was analyzed using FCAP Array™ Software (BD). The flow cytometry data were exported in FCS file format v3 and analyzed using FlowJo software (Treestar Inc.). The graphics and statistics were generated using Prism v5 software (Graph Pad).

### Peptide synthesis

Three peptides from extracellular domains of HER2/neu (HER2/neu_42–56_, _98–114_, and _328–345_) and three peptides from intracellular domains (HER2/neu_776—790_, _927—941_, and _1166–1180_) [17], HLA-A*02:01 restricted viral (NLVPMVATV – CMV_PP65_, GLCTLVAML – EBV_280–288_ BMFL1, and GILGFVFTL – influenza M1), and TAAs HER2/neu_369–377_ (KIFGSLAFL), HER2/neu_689–697_ (RLLQETELV), HER2/neu_435–443_ (ILHNGAYSL), and NY-ESO1_157–165_ (SLLMWITQV) peptides were generated through solid phase peptide synthesis (viral peptides were purchased to 21st Century Biochemicals, CPC Scientific) and TAAs peptides were synthesized at the Fundación Instituto de Inmunología de Colombia - FIDIC), with purity >85 % analyzed by mass spectrometry. The lyophilized peptides were dissolved in DMSO and diluted in PBS to a working concentration of 2 mM each.

### Tetramer staining

Biotinylated HLA-A*02:01 tetramers were synthesized by CPL at the Lawrence Stern Laboratory, University of Massachusetts Medical school. The tetramers were labeled with streptavidin-PE or streptavidin Alexa 700 (Invitrogen™ Life Technologies) at a 4:1 molar ratio in a stepwise addition before use. For tetramer staining process, cells were labeled with 2 μg/mL of corresponding tetramers during 2 h at 37 °C, followed by the addition of the corresponding antibody cocktail for surface markers added as described above. In Fig. 2, CD8+ T cells were labeled with HER2/neu_369–377_ APC MHC dextramer kindly gifted by Immudex (Copenhagen – Denmark).

### TCR Vβ quantification and CDR3 sequence

To compare the 24 families of TCR Vβ repertoire in tetramer positive and negative CD8+ T cells, ex vivo PBMCs were stained with HER2/neu_369–377_ biotin tetramer labeled with streptavidin Alexa fluor 700 as described above in 8 different tubes, followed by TCR Vβ family label using IOTest Beta Mark TCR V Kit (Beckman Coulter, Pasadena, United States) with each antibody cocktail (vials A to H) and anti-CD8 PE-Texas Red (eBiosciences, San Diego, United States); a minimum of 5×10^4^ CD8+ T cells were acquired, and the percentage of each family in HER2/neu specific CD8+ T cells was analyzed with FlowJo software (Treestar, Ashland, United States). For CDR3 sequences, 2×10^7^ PBMCs collected before and after anti-TTx were used to obtain genomic DNA using Wizard® Genomic DNA Purification Kit (Promega Corp., Madison, United States) following manufacturer’s protocol. For the CDR3 sequencing of TILs, two tumor slices (3 μm thick each) were obtained from tumor resection by surgery of the MCC-002 patient fixed-formalin paraffin embedded (FFPE). The DNA from the FFPE sample (extracted by ImmunoSEQ) and DNA from the two PBMCs samples were verified and then sequenced by ImmunoSEQ service (Adaptive Biotechnologies, Seattle, United States), raw data can be found in Additional file 3: Table S1.

## Results

### The anti-tumor therapy reestablishes T cell responsiveness

**Fig. 1:**
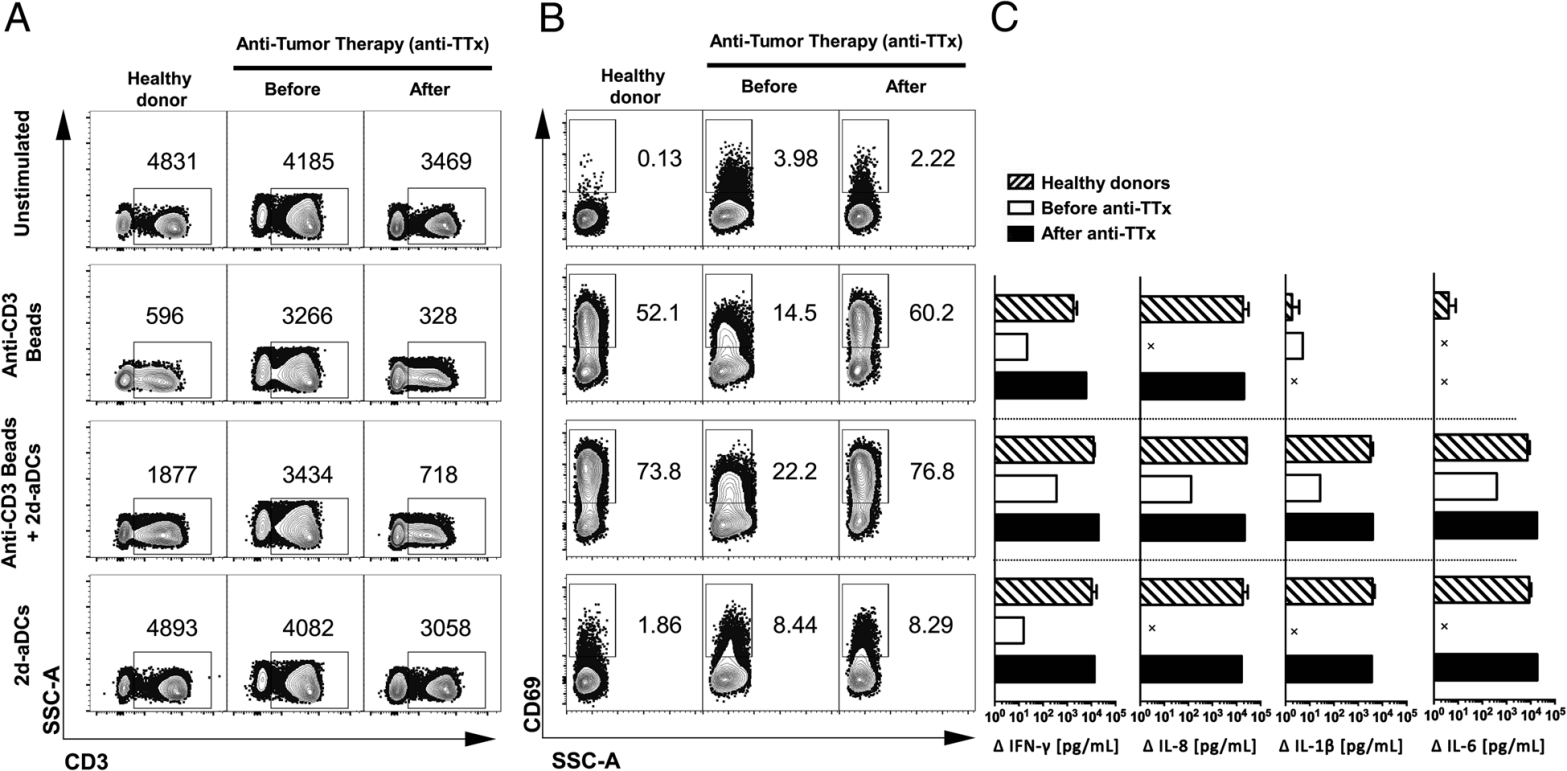
Chemotherapy restores deficient immune response in breast cancer patient. **a**. Representative contour plots of SSC-A vs CD3 in Lymphocyte cells (by SSC-A vs FSC-A) after 72 h of polyclonal stimulation with anti-CD3/CD28/CD2 micro beads, 2d-aDCs or a combination of micro beads in addition to 2d-aDCs in healthy donors (*n* = 12), and breast cancer patients (*n* = 8) (before and after anti-TTx), numbers inside correspond to MFI of CD3 in lymphocyte gate (SSC-A vs FSC-A). **b**. Contour plots of CD69 percentage expression in T cells (CD3+) as in panel A, numbers correspond to the percentage of CD69 expressing cells in CD3+ T cells. **c**. Delta (from left to right) of IFN-γ, IL-8, IL-1β, and IL-6 cytokines relative to unstimulated control in healthy donors (Dashed bars) (*n* = 6) and MCC-002 breast cancer patient (before and after anti-TTx white and black bars respectively), bars show SEM. X: not detected. Results of experiments presented in panel **c** are representative of three performed

Experimental evidence suggests that cancer patients - similar to what has been described in some chronic viral infections - experience a reduction in T cell function [4, 18], in order to determine the effect of anti-TTx on the responsiveness of the T cell compartment, the capacity in MCC-002 patient’s T cells to respond either to a TCR stimulation or to a pro-inflammatory stimuli was compared in PBMCs from blood samples taken before and after anti-TTx (see patients details in Additional file 1: Figure S1). For this, we established first an in vitro assay using PBMCs from healthy individuals stimulated with a mixture of anti-CD3, anti-CD28 and anti-CD2 conjugated to micro beads in order to measure the degree of TCR internalization and expression of CD69, CD25, and CD154 on T lymphocytes of normal individuals. The response to TCR stimulation in T cells from healthy donors was then compared with those from the patient before and after anti-TTx. TCR internalization in response to stimulation was determined by the mean fluorescence intensity (MFI) of CD3. This measurement showed that patient’s T cells prior therapy had a limited capacity to internalize the TCR compared to cells from healthy donors. Notably, the TCR internalization was 10 times higher after anti-TTx than before therapy (MFI values before and after anti-TTx were 3266 and 328 respectively – Fig. 1a) suggesting that this function was recovered after anti-TTx. The low capacity of TCR internalization observed in cells prior anti-TTx was accompanied by a limited expression of CD69, CD25, and CD154 in response to TCR stimulation compared with surface levels of these molecules on stimulated cells from healthy donors (Fig. 1b and Additional file 4: Figure S3 respectively). Likewise, to TCR internalization after anti-TTx the cells recovered the expression of these three molecules to levels similar to those found in cells from healthy donors (Fig. 1a, b and Additional file 4: Figure S3). Interestingly, the secretion of IFN-γ, IL-8, IL-1β, and IL-6 in response to TCR stimulation showed that before anti-TTx the cells had a deficit to secrete these cytokines (Fig. 1c), however, after anti-TTx the T cell responsiveness measured in terms of IFN-γ and IL-8 secretion was recovered (Fig. 1c). Finally, whereas induction of 2d-aDCs *in situ* in cells from healthy individuals showed in culture supernatants a high concentration of IFN-γ, IL-8, IL-1β, and IL-6 that was not observed in cells of the patient before treatment, the induction *in situ* of 2d-aDCs in cells after anti-TTx elicited levels of these cytokines similar to those detected in cells from healthy individuals (Fig. 1c). Altogether, these results indicate that anti-TTx in this patient restores the ability of T cells to respond to TCR stimulation and to a pro-inflammatory stimulus provided by 2d-aDCs.

### 2d-aDCs helps revealing responsiveness of tumor specific CD8+ T cells induced by anti-TTx

**Fig. 2:**
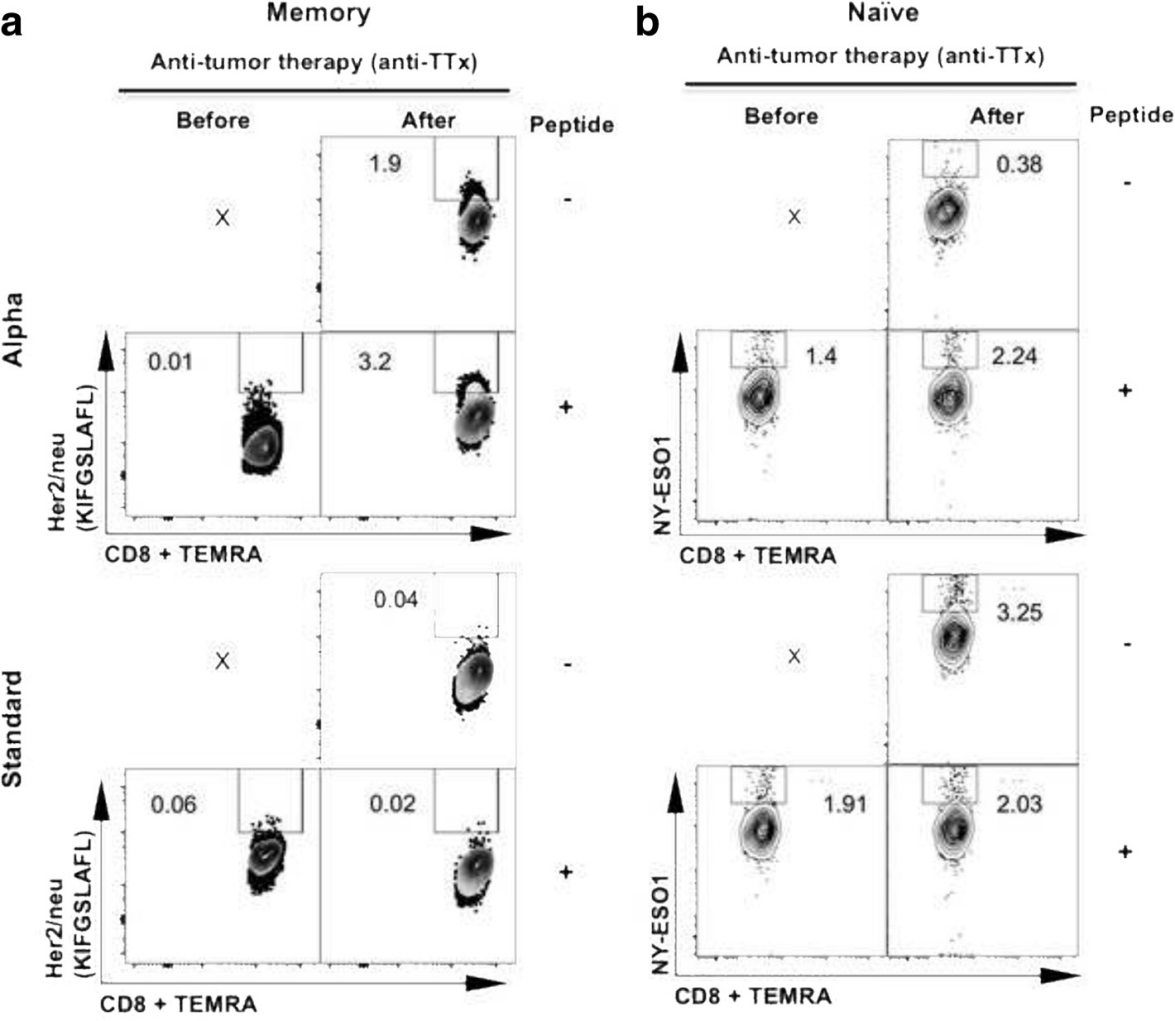
2d-aDCs induce expansion and activation of CD8+ NY-ESO1 and HER2-specific T cells in a breast cancer patient after anti-TTx. **a**. Percentage of TEMRA HER2/neu-specific CD8+ T cells subsequent to induction of *in situ* 2d-aDCs or 2d-stDCs in total PBMCs (MCC-002) induced for 6 days comparing samples obtained before (left column) and after anti-TTx (right column) pulsed with HER2/neu_369–377_ peptide. **b**. Percentage of TEMRA NY-ESO 1-specific CD8+ T cells from patient MCC-002 derived from naïve CD8+ T cells in co-culture with 2d-aDCs or 2d-stDCs-pulsed/unpulsed with NY-ESO 1_157–165_ and stimulated with DCs for 14 days and boosted with corresponding DCs for 6 additional days, comparing samples obtained before (left column) and after anti-TTx (right column). X: not done. Results of experiments presented in panels **a** and **b** are representative of two performed

To explore the effect of anti-TTx in favoring the responsiveness of the T cell compartment in breast cancer patients, the expansion of CD8+ memory T cells [7] that recognize HLA-A*02:01 restricted epitopes from HER2 and NY-ESO 1 was analyzed in PBMCs before and after anti-TTx in our patient stimulated with 2d-aDCs or 2d-stDCs as control APCs using two different in vitro systems (Additional file 2: Figure S2). The first in vitro system is based on the method originally described by Martinuzzi et al., [13] to study the responsiveness of antigen specific memory T cells in peripheral blood (Additional file 2: Figure S2A) patient’s PBMCs were stimulated for six days with either 2d-stDCs or 2d-aDCs derived *in situ* with cytokines and pulsed with a pool of three HER2/neu and one NY-ESO 1 HLA-A*02:01 restricted epitopes. We did not observe ex vivo difference in the percentage of HER2- or NY-ESO 1-specific memory T cells among total CD8+ cells in samples obtained before and after therapy (data not shown). In contrast, in cultures stimulated with peptide-pulsed 2d-aDCs, the percentage of HER2/neu_369–377_ (KIFGSLAFL) tetramer-positive TEMRA CD8+ T cells (CD62-L^low^/CD45RO^low^/CD45RA^high^) was higher after than before anti–TTx (3.2 % and 0.01 % respectively). This expansion after treatment was not observed when the cultures were stimulated with peptide-pulsed 2d-stDCs (percentages before and after anti-TTx were 0.06 % and 0.02 % respectively) (Fig. 2a). These results suggest that 2d-aDCs are more efficient than 2d-stDCs in evidencing the responsiveness of anti-tumor memory T cells elicited by anti-TTx. We failed to detect in PBMCs from this patient CD8+ memory T cells specific for NY-ESO 1_157–165_ epitope (SLLMWITQV) using 2d-aDCs or 2d-stDCs (Additional file 2: Figure S2A and data not shown).

The second in vitro system is based on methodology used by Moser et al., to estimate the repertoire of antigen specific naïve T cells present in peripheral blood [14]. To do this, naïve CD4+ and CD8+ T cells obtained from blood samples before and after therapy were primed independently with either 2d-stDCs or 2d-aDCs pulsed with HER2/neu_369–377_ or NY-ESO 1_157–165_ peptides. After 14 days of priming, the cultures were boosted with the corresponding DCs pulsed with peptide for one additional week (Additional file 2: Figure S2B). Stimulation of naïve T cells with peptide pulsed DCs showed that naïve T cells specific for NY-ESO 1 (SLLMWITQV) HLA-A*02:01 epitope from samples after anti-TTx were primed and boosted more efficiently by 2d-aDCs than naïve T cell preparations obtained before therapy (2.24 % vs. 1.4 %, respectively). 2d-stDCs did not induce the expansion of NY-ESO 1-specific CD8+ T cells compared with unpulsed DCs (2.03 % and 3.25 %, respectively) (Fig. 2b). After several attempts, no expansion of HER2/neu_369–377_ (KIFGSLAFL)-specific CD8+ T cells using this in vitro system was observed (data no shown). Thereafter, we quantified IFN-γ and TNF-α cytokine secretion in both CD8+ and CD4+ T cell culture supernatants (delta of peptide-pulsed DCs minus unpulsed DCs). IFN-γ and TNF-α were secreted in higher concentrations by patient’s cells after anti-TTx stimulated with peptide-pulsed 2d-aDCs. In contrast, we observed that in patient’s naïve T cells (CD8+ or CD4+), 2d-stDCs induced similar levels of cytokine secretion in samples obtained before or after anti-TTx (Additional file 5: Figure S4). These results suggested that after anti-TTx, 2d-aDCs successfully prime and boost the naïve repertoire of anti-tumor CD4+ and CD8+ T cells present in the peripheral blood of this breast cancer patient.

### Anti tumor-therapy induces the expansion and activation of tumor specific effector CD8+ T cells

**Fig. 3:**
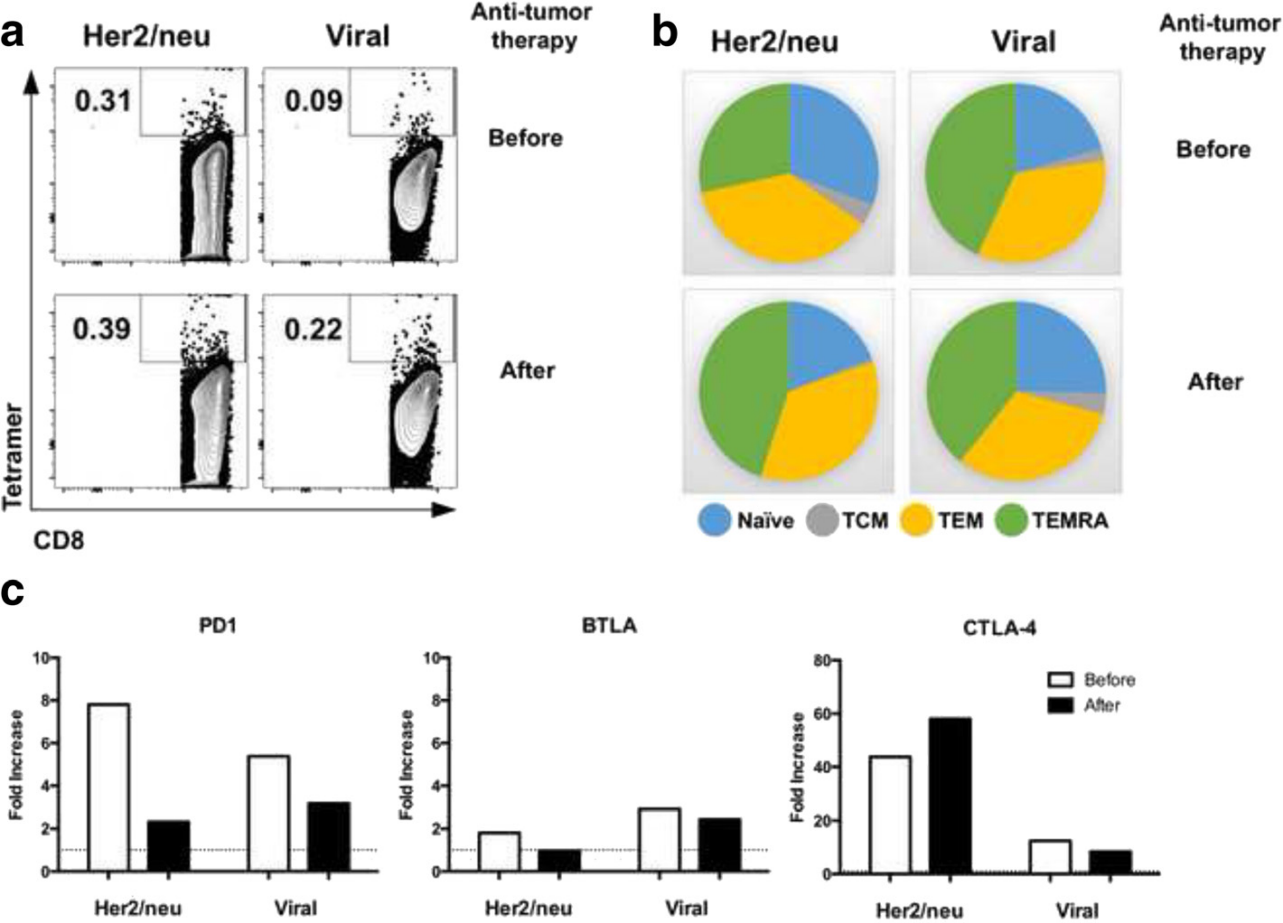
Anti-TTx induces enrichment of CTLA-4 in tumor specific T cells. **a**. Expression of tetramer positive (pool of Her2/neu or viral tetramers) in CD8+ T cells after 6 days of stimulation with aDCs derived *in situ* and pulsed with a combination of HLA-A*02:01 restricted peptides (TAA peptide pool HER2/neu_369–377_, HER2/neu_689–697_ and HER2/neu_435–443_, or viral peptide pool CMV_PP65_, EBV_280–288_, and FLU_58–66_). **b**. Pie chart of naïve and memory sub-populations distribution in tetramer positive CD8+ T cells (TAA or viral specific) after stimulation with pulsed 2d-aDCs before and after anti-TTx (Blue; naïve, Grey; TCM, yellow; TEM; and green TEMRA). **c**. Fold increase of inhibitory receptors (PD1, BTLA, and CTLA-4) expression between tetramer positive over tetramer negative CD8+ T cells after stimulation with peptide pulsed 2d-aDCs, obtained before (white bars) and after (black bars) anti-TTx

To assess efficacy of anti-TTx in fostering the expansion of tumor specific T cells, we compared in blood samples of this breast cancer patient before and after anti-TTx, the frequency and the memory phenotype of CD8+ T cells specific for several HLA–A*02:01 restricted tumor epitopes (TAAs): Initially, we evaluate the expansion of Her2/neu specific T cells in total PBMC in response to 2d-aDCs pulsed with a pool of three different HLA-A*02:01 restricted peptides (HER2/neu_369–377_ KIFGSLAFL, HER2/neu_689–697_ RLLQETELV, and HER2/neu_435–443_ ILHNGAYSL) and compared with the expansion of viral specific T cells (cytomegalovirus CMV pp65_495–503_ NLVPMVATV; Influenza Matrix protein 1 FLU_58–66_ GILGFVFTL, and EBV BMLF1 protein EBV_280–288_ GLCTLVAML). We evidence a small expansion of TAA and viral specific CD8+ T cells after 6 days of stimulation with peptide pulsed 2d-aDCs in PBMCs obtained after anti-TTx (Fig. 3a), in contrast, we observe a different distribution of naïve and memory sub-populations between TAA vs viral specific T cells, in response to peptide pool viral specific T cells have a high proportion of TEM and TEMRA phenotype, compared to TAA specific T cells before anti-TTx. The phenotype of TAA specific T cells change in response to anti-TTx, with a similar distribution of viral specific T cells (Fig. 3b). To further characterize the immune-phenotype of tetramer positive CD8+ T cells after in vitro expansion with 2d-aDCs, we evaluated the expression of three different inhibitory receptors (PD1, BTLA and CTLA-4) on tetramer positive cells [19] and compared with the expression of these receptors in total CD8+ T cells; we found a substantial fold increase only in CTLA-4 expression in TAA specific CD8+ T cells after compared to before anti-TTx (43 before- to 58 after anti-TTx) with no major changes in cells specific for viral antigens or the expression of PD1, or BTLA (Fig. 3c).

**Fig. 4:**
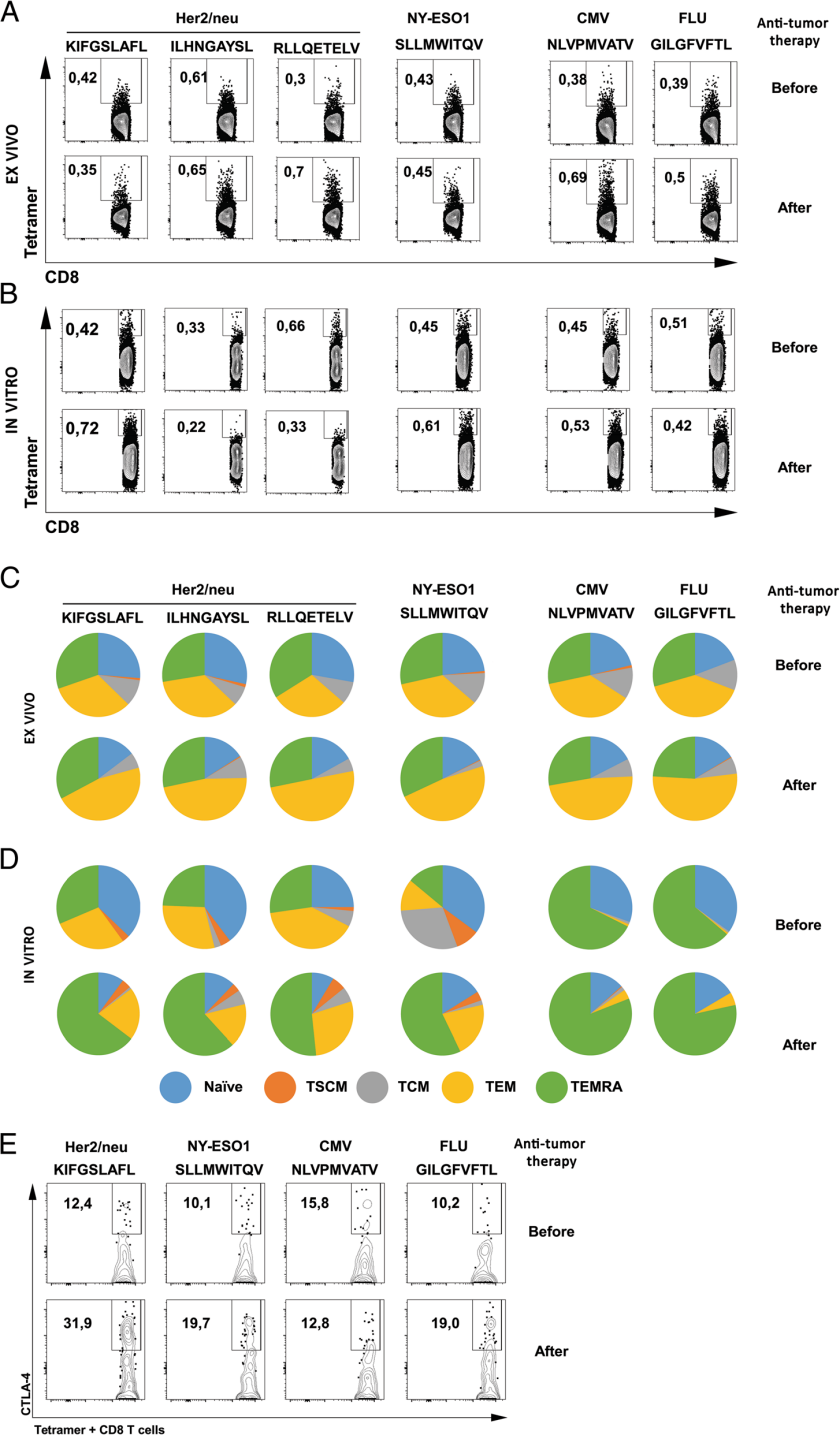
Anti-TTx induces the expansion, differentiation and CTLA-4 expression in Her2/neu_369–377_ tumor specific CD8+ T cells. **a**. Representative contour plots of tetramer specific CD8+ T cells staining for HER2/neu_369–377_ (KIFGSLAFL), HER2/neu_689–697_ (RLLQETELV), HER2/neu_435–443_ (ILHNGAYSL) and NY-ESO 1_157–165_ (SLLMWITQV), and two tetramers for viral antigens (NLVPMVATV – CMV_PP65_, and GILGFVFTL – influenza M1 FLU_58–66_), stained ex vivo in PBMCs from a breast cancer patient before and after anti-TTx in purified CD8+ T cells. The number inside the plots represents the percentage of tetramer-specific CD8+ T cells. **b**. Representative contour plots of tetramer specific CD8+ T cells after 7 days of in vitro stimulation of PBMCs with the corresponding peptide pulsed 2d-aDCs, the number inside the plots represents the percentage of tetramer-specific CD8+ T cells. Results of experiments presented in panels **a**, and B are representative of two performed. **c**. and **d**. Representative pie charts of ex vivo (**c**) or after in vitro PBMC stimulation (**d**), as described in **a** and **b**, with the percentage of the phenotype of naïve and memory sub-populations in CD8+ tetramer positive T cells for four TAAs and two viral HLA-A*02:01 peptides before and after anti-TTx. Blue; naïve, Orange; TSCM, Grey; TCM, yellow; TEM; and green TEMRA. Results of experiments presented in panels **c** and **d** are representative of two performed. **e**. Representative contour plots of CTLA-4 vs CD8 in tetramer positive CD8+ T cells for HER2/neu_369–377_ (KIFGSLAFL) and NY-ESO1_157–165_ (SLLMWITQV) tetramer + CD8+ T cells for two viral antigens (NLVPMVATV – CMV_PP65_, and GILGFVFTL – influenza M1 FLU_58–66_), in PBMCs stimulated with *in situ* 2d-aDCs from PBMCs obtained before and after anti-TTx as described in **a** and **b**. Results of experiments presented are representative of two performed

To evaluate the specificity of the anti-tumor immune response against each TAA elicited by anti-TTx, the profile of tetramer positive CD8+ T cells specific to four individual tumor epitopes was measured ex vivo and in vitro (Fig. 4a left panels). This profile was compared to that of tetramer positive CD8+ T cells specific for three HLA-A*02:01 restricted viral epitopes CMV pp65_495–503_; FLU_58–66_ (Fig. 4a right panels) and EBV _280–288_ (data not shown). There were not major differences ex vivo in the percentages of CD8+ tetramer positive cells specific for tumor or viral antigens in samples obtained before and after anti-TTx (Fig. 4a). On the other hand, when PBMCs were stimulated with *in situ* derived 2d-aDCs in the presence of each TAA, the frequency of tetramer positive CD8+ T cells showed some increase in response to HER2/neu_369–377_ (KIFGSLAFL) epitope (from 0.42 % before to 0.72 % after anti-TTx) and to lower extent in response to NY-ESO1 (from 0.45 % to 0.61 % before vs. after anti-TTx). Neither before not after the anti-TTx, CD8+ T cells specific for viral antigens exhibited significant expansion upon stimulation in vitro with 2d-aDCs pulsed with each viral epitope (Fig. 4b).

The analyses ex vivo of the distribution of naïve and memory T cell sub-populations within tetramer positive CD8+ T cells specific for tumor or viral antigens did not show major differences among samples obtained before or after anti-TTx (Fig. 4c right and left panels). As expected a remarkable expansion of TEMRA CD8+ T cells was observed in samples before and after anti-TTx upon stimulation of the cells in vitro for six days with CMV, FLU (Fig. 4d right panels) and EBV (data not shown) viral peptides. That similar responsiveness of CD8+ T cells specific to tumor antigens was observed after individual in vitro stimulation with four different tumor epitopes only in samples obtained after anti-TTx and was not evident in cells before anti-TTx (Fig. 4d left panels), lead us to argue that the anti-TTx in this patient efficiently promotes the responsiveness of tumor specific effector CD8+ T cells (TEMRA). Based on the increase of CTLA-4 expression in TAA specific CD8+ T cells after anti-TTx, we evaluated the expression of CTLA-4 on tetramer positive cells; we found a substantial increase in the percentage of CTLA-4 expression in HER2/neu_369–377_; NY-ESO 1 and FLU specific CD8+ T cells after compared to before anti-TTx (12.4 % before- to 31.9 % after anti-TTx; 10.1–19.7 % and 10.2–19.0 % respectively) with no major changes in cells specific for CMV (15.8 % prior- to 12.8 % post-treatment) (Fig. 4e).

### Expansion of TAA specific CD8+ T cells after anti-tumor therapy correlates with the T cell repertoire of tumor infiltrating lymphocytes

**Fig. 5:**
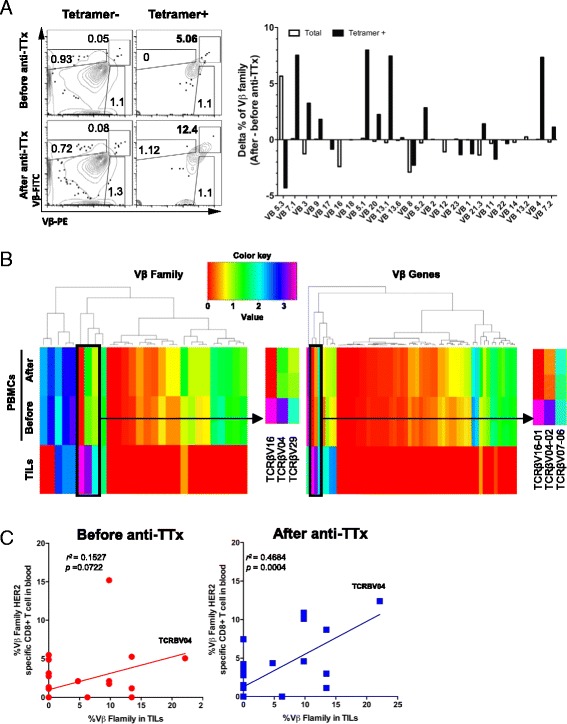
Increased correlation of TCR Vβ families between tumor infiltrating lymphocytes and HER2/neu tetramer CD8+ T cells after chemotherapy. **a**. Dot plot example of an ex vivo flow cytometry analysis of TCR-Vβ families 13.2, 4 and 7.2 in CD8+ T cells HER2/neu_369–377_ tetramer negative vs. tetramer positive from before and after anti-TTx cells, each gate represents one TCR-Vβ family, PE positive correspond to Vβ13.2, the double positive cells corresponds to family Vβ4, and FITC positive corresponds to family Vβ7.2, numbers correspond to percentage of each family, left; Delta of the percentage of each of 24 families (after minus before therapy) in total CD8+ T cells (empty bars) or tetramer positive CD8+ T cells (black bars), right. **b**. Heat map of sequenced TCRs from total PBMCs (before and after anti-TTx) and Tumor Infiltrating Lymphocytes (TILs) from FFPE tumor tissue slice. Arrows points insert of highly expressed of TCR Vβ families and genes in TILs. **c**. Correlation plots of Vβ families of HER2/neu_369–377_ tetramer positive cells (red corresponds to before anti-TTx sample, and blue corresponds to after anti-TTx sample) analyzed by flow cytometry vs. the percentage of Vβ families obtained from TILs. Pre-chemotherapy r^2^: 0.164, *p* value: 0.017, post-chemotherapy r^2^: 0.477, *p* value: <0.0001 (linear regression analysis)

To assess changes in the repertoire of TAA specific CD8+ T cells in peripheral blood induced by anti-TTx and their relationship with tumor infiltrating lymphocytes, we compared to ex vivo by FC the TCR-Vβ repertoire of HER2/neu_369–377_ tetramer positive CD8+ T cells present in PBMCs from the patient before and after anti-TTx. The percentage of 24 Vβ families in tetramer positive and tetramer negative CD8+ T cells before and after anti-TTx is summarized in Fig. 5a right panel. This result showed the enrichment in tetramer positive cells after anti-TTx of the Vβ families Vβ7.1, Vβ9, Vβ5.1, Vβ20, Vβ13.1, Vβ5.2, and Vβ04 (Fig. 5a). The specificity and the frequency of T cells is generated by somatic rearrangement of TCR genes and mainly focused on the CDR3 region. This has been evaluated by spectratyping in combination with family quantification of TCR-Vβ by FC in cancer patients [20] making these systems useful for T cell identification, but recent techniques can sequence the CDR3 region allowing the quantification of each TCR at a gen level [21]. This technology in combination with TAA MHC-class I tetramers can be relevant for the evaluation of TAA specific T cells in response to anti-TTx. In order to establish the identity of these TCRs we sequenced the CDR3 region and estimated the copy number of encoding transcripts in PBMCs samples obtained before and after anti-TTx from our breast cancer patient. To evaluate the tumor infiltration capacity of these cells we performed a similar analysis in tumor infiltrating lymphocytes (TILs) using fixed-formalin paraffin embedded (FFPE) tumor slices. Figure 5b shows the profile of TCR Vβ families (left heat map) and TCR Vβ genes (right heat map) in blood before and after anti-TTx and in TILs with those at the highest frequencies highlighted in black squares. Whereas the heat map of Vβ families and defined genes expressed by T cells in blood samples before and after therapy evidenced great variability, in TILs, Vβ16 and Vβ04 families (Vβ16-01 and Vβ04-02 genes) were present at high frequency (Fig. 5b heat map lower rows). Finally, in trying to correlate specificity of TILs with expansion of CD8+ T cells specific for HER2/neu_369–377_ detected in blood by FC, we correlated frequency of Vβ families expressed by tetramer positive cells (Fig. 5a) with the frequency of the same Vβ families in TILs detected by the TCR CDR3 sequence (Fig. 5b). Interestingly, we found a better correlation (*r*^2^) of Vβs expressed by TILs with that of HER2/neu_369–377_ specific CD8+ T cells in blood samples after than before anti-TTx (*r*^2^: 0.47 and *r*^2^: 0.15 respectively) with a high correlation of Vβ04 (Fig. 5c). These results suggest that the anti-TTx induced an increase of HER2/neu_369–377_ specific CD8+ T cells capable of infiltrating the tumor and that this increase can be detected in the peripheral blood of this patient.

## Discussion

Previous results obtained by our group showed that alpha DCs generated in two days (2d-aDCs) has a mature phenotype similar to those generated by Mailliard et al., in seven days [12] and also similar to standard DCs generated in seven (7d-stDCs) [12, 22] or two (2d-stDCs) days [23, 24] (DB et al., manuscript in preparation). One of the major functional characteristics of aDCs is the secretion of IL-12 and we found that 2d-aDCs secrete IL-12 more efficiently than 2d-stDCs (data not shown) suggesting that 2d-aDCs are not in an exhausted state [25]. The production of IL-12 by DCs is of paramount importance for plasma cell differentiation; for antibody production promoted by follicular CD4+ T-helper cells (TFH) [26] and for the generation of anti-tumor specific CD8+ T cells [27–29] both in vitro [30] and in vivo [31], hence, IL-12 production by DCs is considered one important requirement for DC-based cancer immunotherapy. In this work, we use 2d-aDCs as a tool for assess Th1 response against TAAs and to monitor the responsiveness of anti-tumor specific T cells induced in cancer patients by anti-TTx.

To evaluate the immune response of the T cell compartment in patients with breast cancer, we established an in vitro method of polyclonal stimulation of T cells to assess the responsiveness of T cells characterized by internalization of CD3, the expression of different activation markers and secretion of Th1 cytokines. This system enabled us to determine that before the anti-TTx, T cells obtained from a breast cancer patient who is free of disease after several years of completion of the anti-TTx, exhibited a pronounced defect in responsiveness to stimuli and that anti-TTx efficiently rescued the sensitiveness of T cells to TCR stimulation. The responsiveness of the T cell compartment to the stimulus elicited by anti-TTx was measured through different immunological readouts such as TCR internalization, surface expression of activation markers and secretion of cytokine that were low in cells before therapy but that in T cells from the patient after anti-TTx reached similar levels to those observed in stimulated T cells from healthy individuals (Fig. 2). These results support the adjuvant effect of the anti-TTx in the T cell compartment of breast cancer patients proposed by others in preclinical studies of cancer mouse models [32]. Based on these observations, we are currently analyzing this phenotype in a series of patients with breast cancer before and after treatment with neo-adjuvant chemotherapy with anthracyclines (doxorubicin) and cyclophosphamide with similar results (DB et al., manuscript in preparation).

In order to evaluate the functional capacity of 2d-aDCs to induce expansion of TAA-specific T cells, two in vitro culture conditions were used (Additional file 2: Figure S2); the first condition was used to determine the degree of expansion and activation of T cells that recognize different TAA present in the pool of memory CD8+ T cells and the second to assess the response of naïve T lymphocytes to priming in vitro with these TAAs. As described previously, the anti-TTx resulted in the recovery of the T cell responsiveness to a polyclonal stimulus. To demonstrate the effect of anti-tumor therapy in response of antigen specific T cells, it was compared in both culture systems the capacity of anti-TTx to favors the expansion in vitro of TAA specific T cells after chemotherapy using as APCs 2d-aDC or 2d-stDCs to stimulate T cells. The results suggest that 2d-aDCs are more efficient than 2d-stDCs in evidencing the expansion of TEMRA CD8+ T cells specific for TAAs favored by anti-TTx (Fig. 2). Notably, the expansion of naïve T cells in post-chemotherapy samples primed in vitro with 2d-aDCs pulsed with TAAs increased IFN-γ and TNF-α in supernatants of CD8 + T cells (Additional file 5: Figure S4). Furthermore, the stimulatory capacity of the anti-TTx on CD8+ T lymphocytes was evidenced in the expansion of TEMRA CD8+ T cells specific for HER2/neu_369–377_ (KIFGSLAFL) epitope only in samples after anti-TTx contrary to the conserved response to viral antigens before and after anti-TTx (Fig. 4). Also HER2/neu specific T cells expressed high levels of CTLA-4 (Fig. 4e), interestingly the expression of CTLA-4 as a biomarker of stimulated CD8+ T cells has been associated with a favorable clinical response in patients in immunotherapy treated with anti-PD-L1 [33]. These results are consistent with the recent analysis of Speiser and colleagues in the sense that the expression on CD8+ T cells of molecules such as PD-1, BTLA and CTLA-4 does not mark necessarily cells with a reduced T cell function but rather mark different subsets of activated T cells linked to positive clinical outcomes [19]. Together these results suggest that in this patient with breast cancer, 2d-aDCs allow the expansion of tumor specific T cells favored by chemotherapy and they could be used to monitor the immunological effects of the anti-TTx on tumor specific T cells. Hence we propose that the observed competence as APCs of 2d-aDCs derived *in situ* from PBMCs will be a useful system for monitoring tumor specific T cells using small volumes of blood in patients with cancer in anti-tumor treatment.

Different studies using next generation sequencing (NGS) show an anti-tumor effect of anti-TTx on the T cell repertoire in different tumors such as lung [34] and colorectal cancer [35]. Combining strategies proposed by Pilch et al., [20] and Robins et al., [21] we analyzed the frequency of TCR Vβ by FC and the identity of TCR CDR3 through NGS to optimize the assessment of TCR repertoire of anti-tumor cells in the patient MCC-002. To establish the identity of TILs we analyzed the Vβ repertoire of TILs in the diagnosis biopsy using FFPE as source of tumor tissue followed by CDR3 sequencing using NGS. The repertoire of TILs was then compared to that obtained ex vivo from blood samples before and after anti-TTx. The results of the analysis of the CDR3 repertoire in the biopsy and in blood before and after therapy lead us to suggest that anti-TTx favors the expansion in peripheral blood of tumor specific T cells that infiltrate the tumor before therapy. Altogether our results suggest that the use of FFPE as a source of tumor tissue combined with NGS and the detection and functional analysis of T cells that recognize TAAs in blood using fluorescent tetramers may be useful to monitor both the dynamics of the traffic of TILs between tumor and peripheral blood and for the assessment of the functional status of these cells in peripheral blood during anti-TTx.

Evaluating to what extent clinical response to cancer is attributable to the activation of anti-tumor T cells induced by chemo- or radiotherapy is currently a matter of interest of numerous clinical studies [36]. Similar to what was shown in patients with colorectal cancer under radiotherapy [37]; in the present study using 2d-aDCs we found that the anti-TTx stimulates CD8+ anti-tumor T cells. The use of these cells as APCs reveals a stimulatory effect of anti-TTx on the responsiveness of CD8+ T cells specific for TAAs in breast cancer patients. The results presented here, were possible by the considerably amount of cells obtained from an ideal patient (Her2/neu+++, HLA-A*02:01), are limited by the number of patients that could be followed up with this deep immunological study. The recovery of the unresponsiveness observed prior anti-TTx is reflected in the improvement in T cell function. As a whole, our results suggest that evidencing the adjuvant effect of the anti-TTx in breast cancer that can be explored in other tumors.

## Conclusions

The anti-tumor therapy benefits the immune response of the treated patient. This benefit was evidenced in vitro by increasing the T cell responsiveness and expanding tumor-specific CD8+ T cells in peripheral blood with a functional phenotype of memory effector cells. This in vitro approach can be implemented in a cohort of breast cancer patients in order to evidence the effect of other chemotherapy regimens that includes anthracyclines, known for inducing immunogenic cell death in tumor cells, and to establish a possible predictive marker for clinical response.

## Abbreviations

2d-aDCs, two-day derived alpha dendritic cells; 2d-stDCs, two-day derived standard dendritic cells; anti-TTx, anti-tumor therapy; APCs, antigen presenting cells; CBA, cytometric bead array; CTL, cytotoxic lymphocytes; DCs, dendritic cells; DMSO, dimethyl sulfoxide; FBS, fetal bovine serum; FC, flow cytometry; FFPE, formalin-fixed paraffin embedded; iDCs, immature dendritic cells; IL, interleukin; moDCs, monocyte derived Dendritic Cells; NGS, next generation sequencing (NGS); PBMCs, peripheral blood mononuclear cells; TAAs, tumor associated antigens; TCM, central memory T cell; TEM, effector memory T cell; TEMRA, effector memory CD45RA+ T cell; TILs, tumor infiltrating lymphocytes; TLR, toll-like receptors; TN, naïve T cell; TSCM, stem cell like memory T cell

## Additional files

Additional file 1: Figure S1. Timeline of MCC-002 treatment. A 63-year-old woman (MCC-002) was diagnosed with invasive ductal breast carcinoma (stage II, T2N3M0) after a suggestive result in a control process by mammography (BIRADS 6). After the realization of the biopsy, the tumor is positive for HER2/neu (C-erb2), and NY-ESO 1, and negative for hormonal receptors (ER and PR), with compromised axillary lymph nodes but no evidence of bone metastasis. This patient was screened for HLA-A2 by flow cytometry that was confirmed by SSP-PCR for HLA-A*02:01. After signed informed consent, we obtained by leukapheresis a preparation of 150 mL buffy coat enriched in peripheral blood mononuclear cells (PBMC). This patient was treated with modified radical mastectomy (MRM) of the left breast including axillary clearance (5 of 21 lymph nodes were compromised with tumor), with no complications. After surgery (September 2008), the patient was treated with 12 weekly doses of paclitaxel (150 mg) and was programmed for one year of treatment with trastuzumab (440 mg every three weeks) and four weeks of radiotherapy (2.5Gy daily). In March 2009, after the fifth dose of trastuzumab and four doses of radiotherapy, the patient experimented acute cardiac failure with a ventricular ejection fraction (VEF) below 30 % (VEF on August 2008 prior surgery was 59 %). Because cardiac toxicity, the chemo- and radio-therapy were suspended. The cardiac failure was managed successfully with spironolactone (50 mg/daily) and furosemide (40 mg/12 h). The follow-up mammographies were negative (BIRADS 2 for the contralateral breast). Finally, 8 months after the suspension of anti-tumor treatment, a second leukapheresis was obtained in order compare prior vs. after anti-TTx the effect of treatment on the immune response using a number of experimental readouts. Currently, MCC-002 maintains with a clinical complete response after 7 years of the anti-tumor therapy suspension controlled with annual mammography. (TIF 437 kb) Additional file 2: Figure S2. Flowchart of the methodology used for cell culture with 2d-aDCs and 2d-stDCs. A. Methodology for *in situ* induction of 2d-aDC and 2d-stDC in total PBMCs (based on the methodology of Martinuzzi et al., [13]). PBMCs were stimulated for 24 h with IL-4 and GM-CSF with peptide(s) and maturated with a combination of proinflammatory cytokines and TLR ligands for the corresponding DCs with or without a new addition of the corresponding peptide(s) for 6 days. B. T cell stimulation scheme based on the methodology described by Moser et al., [14]. Briefly, 2d-aDCs and 2d-stDCs were derived from monocytes and cultured with purified autologous naïve CD4+ or CD8+ T cells for 14 days and re-stimulated for 6 days with peptide (5 μM)-pulsed 2d-aDCs or 2d-stDCs. (TIF 308 kb) Additional file 3: TCR raw data sequence from PBMCs obtained before and after therapy and TILs obtained from FFPE tumor. (XLSX 27054 kb) Additional file 4: Figure S3. Expression recovery of CD25 and CD154 in T cells after chemotherapy. Fold percentage of CD25 (left) and CD154 (right) in CD3 positive T cells relative to un-stimulated PBMCs as described in Fig. 5 in healthy donors (Dashed bars) (*n* = 3) and a breast cancer patient (pre and post chemotherapy white and black bars respectively), bars show SEM. Results of experiments presented are representative of three performed. (TIF 175 kb) Additional file 5: Figure S4. High IFN-γ and TNF-α secretion from CD8+ and CD4+ T cells after anti-TTx when stimulated with 2d-aDCs. Delta of IFN-γ and TNF-α concentration in pg/mL (pulsed minus unpulsed DCs culture) in the supernatants of CD8+ and CD4+ T cells subsequent to stimulation for 14 days, with boosting for 6 days in samples before and after anti-TTx with 2d-aDCs or 2d-stDCs pulsed with HER2 peptides. Results of experiments presented are representative of two performed, x = data not detected. (TIF 317 kb)
