# Supplementary figures and images for: Chemotherapy and radiation therapy elicits tumor specific T cell responses in a breast cancer patient
Source: BMC Cancer. 2016 Aug 3;16:591. doi: 10.1186/s12885-016-2625-2 (PMC4971722; doi:10.1186/s12885-016-2625-2)

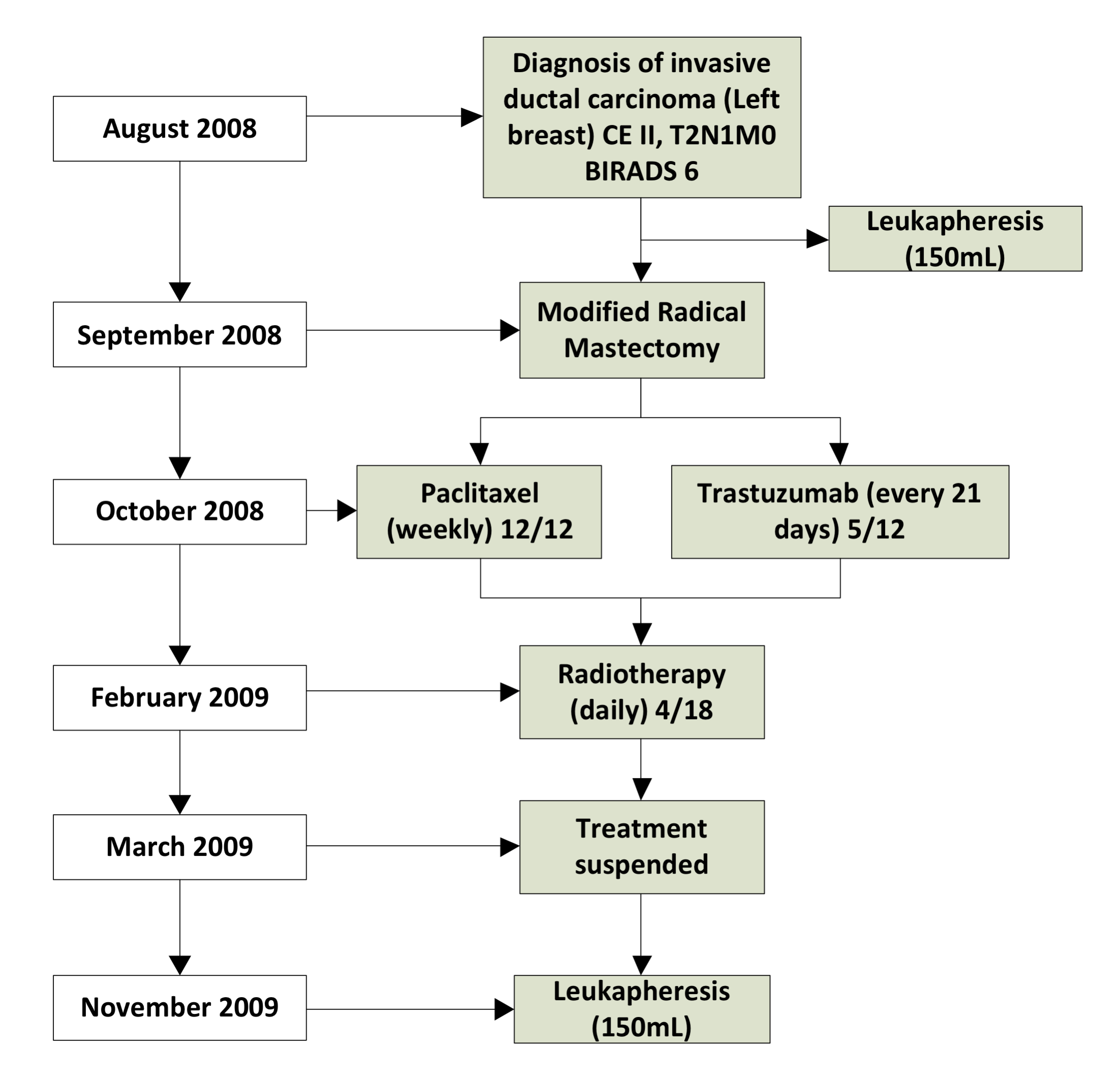

Supplement: Additional file 1: Figure S1. — Timeline of MCC-002 treatment. A 63-year-old woman (MCC-002) was diagnosed with invasive ductal breast carcinoma (stage II, T2N3M0) after a suggestive result in a control process by mammography (BIRADS 6). After the realization of the biopsy, the tumor is positive for HER2/neu (C-erb2), and NY-ESO 1, and negative for hormonal receptors (ER and PR), with compromised axillary lymph nodes but no evidence of bone metastasis. This patient was screened for HLA-A2 by flow cytometry that was confirmed by SSP-PCR for HLA-A*02:01. After signed informed consent, we obtained by leukapheresis a preparation of 150 mL buffy coat enriched in peripheral blood mononuclear cells (PBMC). This patient was treated with modified radical mastectomy (MRM) of the left breast including axillary clearance (5 of 21 lymph nodes were compromised with tumor), with no complications. After surgery (September 2008), the patient was treated with 12 weekly doses of paclitaxel (150 mg) and was programmed for one year of treatment with trastuzumab (440 mg every three weeks) and four weeks of radiotherapy (2.5Gy daily). In March 2009, after the fifth dose of trastuzumab and four doses of radiotherapy, the patient experimented acute cardiac failure with a ventricular ejection fraction (VEF) below 30 % (VEF on August 2008 prior surgery was 59 %). Because cardiac toxicity, the chemo- and radio-therapy were suspended. The cardiac failure was managed successfully with spironolactone (50 mg/daily) and furosemide (40 mg/12 h). The follow-up mammographies were negative (BIRADS 2 for the contralateral breast). Finally, 8 months after the suspension of anti-tumor treatment, a second leukapheresis was obtained in order compare prior vs. after anti-TTx the effect of treatment on the immune response using a number of experimental readouts. Currently, MCC-002 maintains with a clinical complete response after 7 years of the anti-tumor therapy suspension controlled with annual mammography. (TIF 437 kb) [file 12885_2016_2625_MOESM1_ESM.tif]

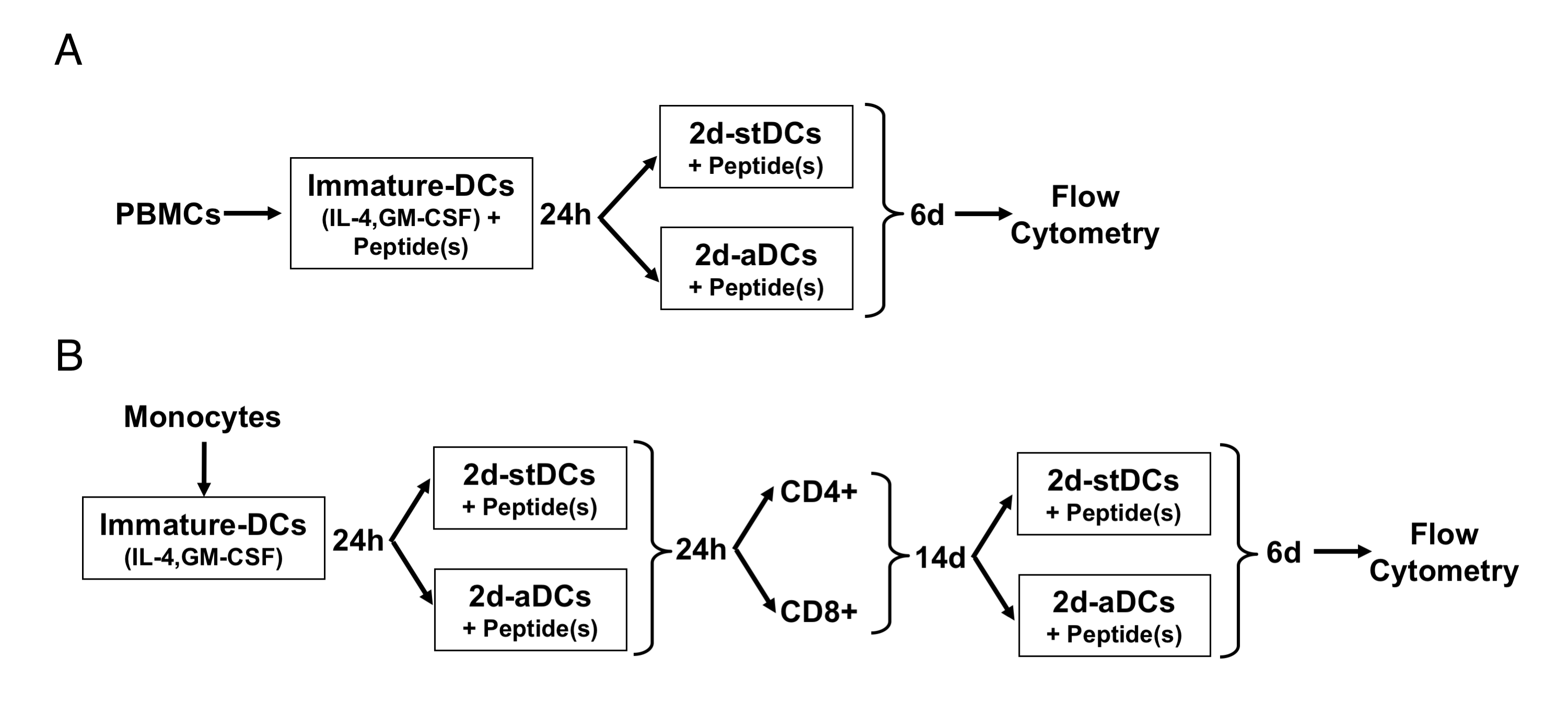

Supplement: Additional file 2: Figure S2. — Flowchart of the methodology used for cell culture with 2d-aDCs and 2d-stDCs. A. Methodology for in situ induction of 2d-aDC and 2d-stDC in total PBMCs (based on the methodology of Martinuzzi et al., [13]). PBMCs were stimulated for 24 h with IL-4 and GM-CSF with peptide(s) and maturated with a combination of proinflammatory cytokines and TLR ligands for the corresponding DCs with or without a new addition of the corresponding peptide(s) for 6 days. B. T cell stimulation scheme based on the methodology described by Moser et al., [14]. Briefly, 2d-aDCs and 2d-stDCs were derived from monocytes and cultured with purified autologous naïve CD4+ or CD8+ T cells for 14 days and re-stimulated for 6 days with peptide (5 μM)-pulsed 2d-aDCs or 2d-stDCs. (TIF 308 kb) [file 12885_2016_2625_MOESM2_ESM.tif]

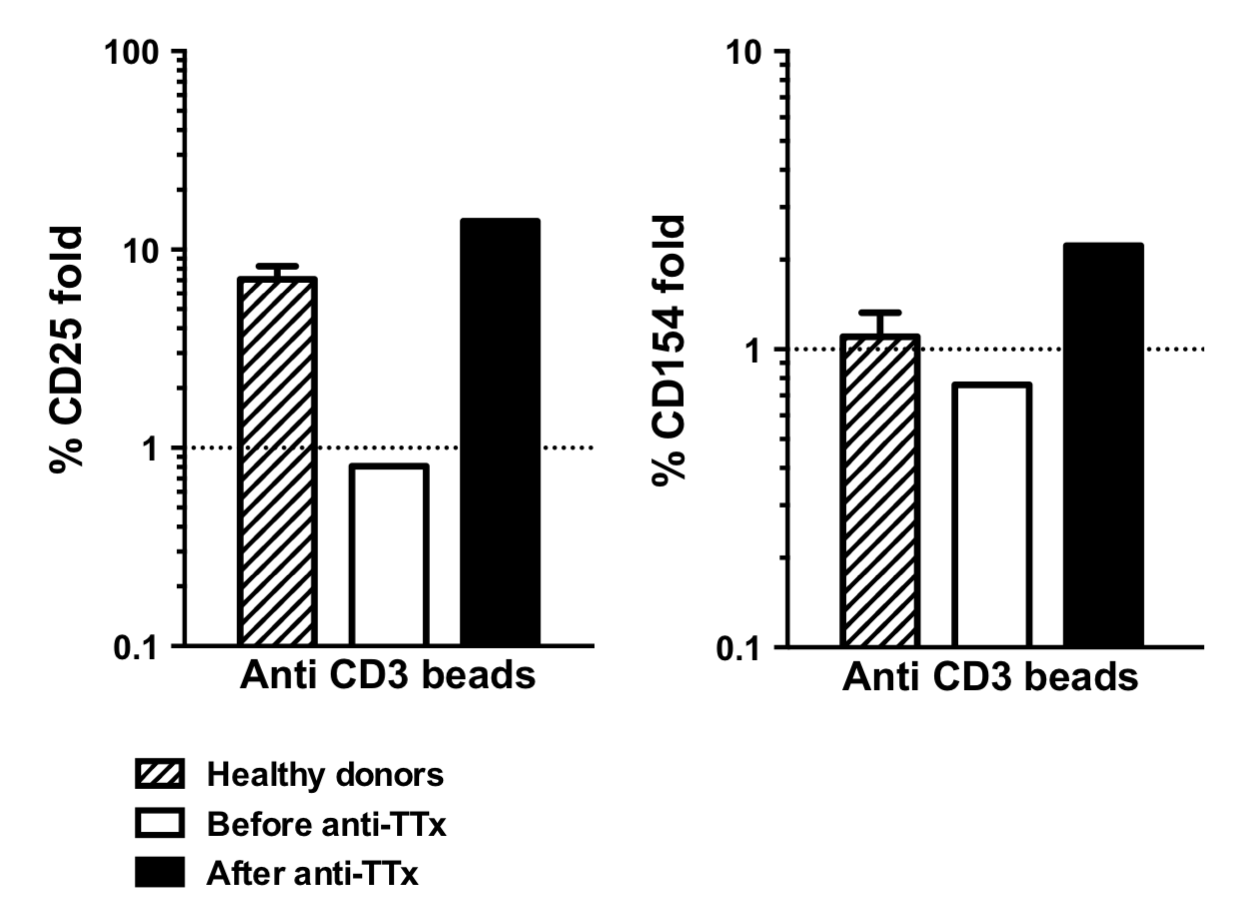

Supplement: Additional file 4: Figure S3. — Expression recovery of CD25 and CD154 in T cells after chemotherapy. Fold percentage of CD25 (left) and CD154 (right) in CD3 positive T cells relative to un-stimulated PBMCs as described in Fig. 5 in healthy donors (Dashed bars) (n = 3) and a breast cancer patient (pre and post chemotherapy white and black bars respectively), bars show SEM. Results of experiments presented are representative of three performed. (TIF 175 kb) [file 12885_2016_2625_MOESM4_ESM.tif]

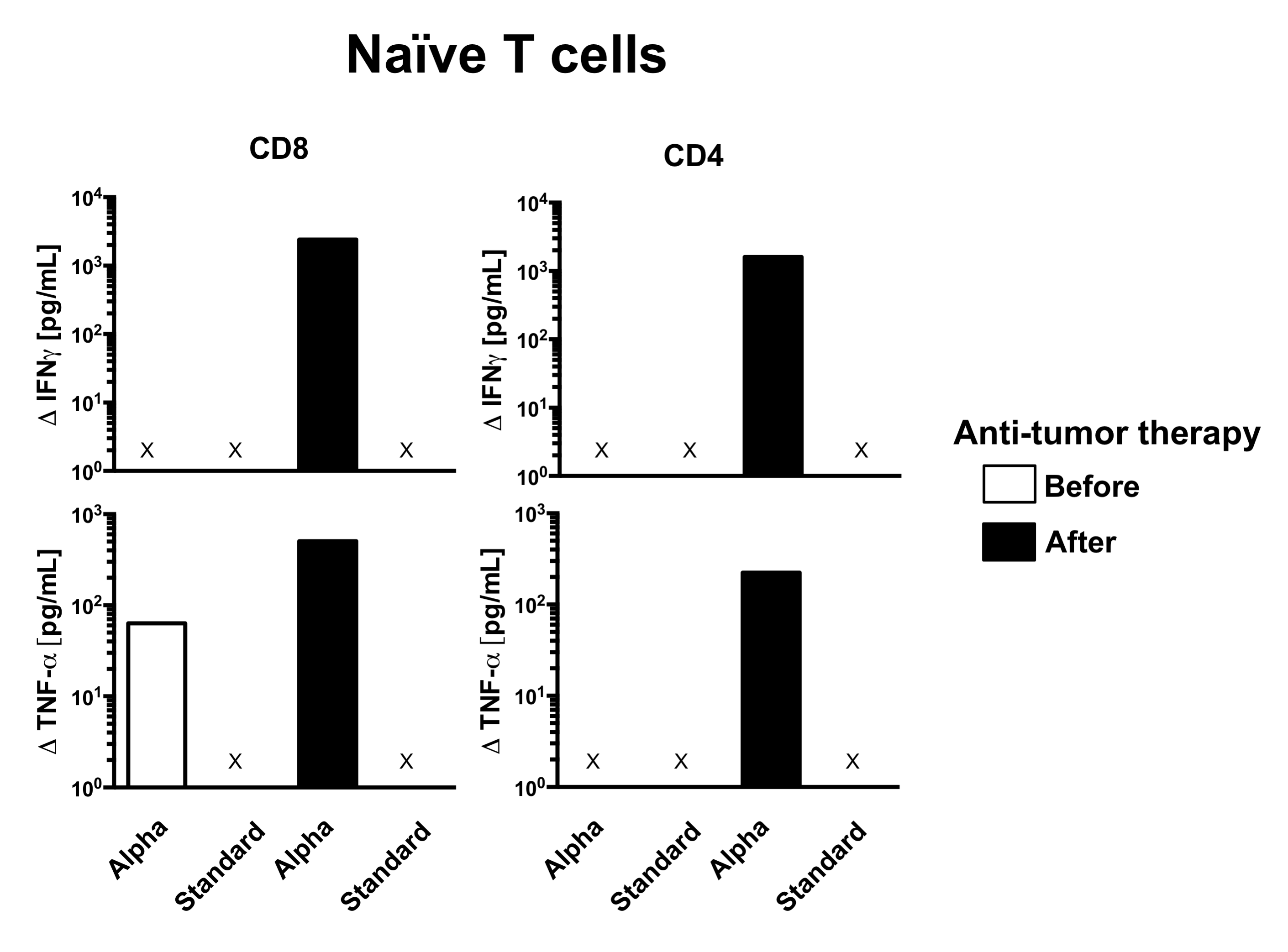

Supplement: Additional file 5: Figure S4. — High IFN-γ and TNF-α secretion from CD8+ and CD4+ T cells after anti-TTx when stimulated with 2d-aDCs. Delta of IFN-γ and TNF-α concentration in pg/mL (pulsed minus unpulsed DCs culture) in the supernatants of CD8+ and CD4+ T cells subsequent to stimulation for 14 days, with boosting for 6 days in samples before and after anti-TTx with 2d-aDCs or 2d-stDCs pulsed with HER2 peptides. Results of experiments presented are representative of two performed, x = data not detected. (TIF 317 kb) [file 12885_2016_2625_MOESM5_ESM.tif]
